# The chemokine CXCL13 is elevated in the cerebrospinal fluid of patients with neurosyphilis

**DOI:** 10.1186/s12987-015-0008-8

**Published:** 2015-05-15

**Authors:** R Dersch, T Hottenrott, M Senel, V Lehmensiek, H Tumani, S Rauer, O Stich

**Affiliations:** Department of Neurology, University Medical Center Freiburg, Breisacher Str. 64, 79106 Freiburg, Germany; Department of Neurology, University of Ulm, Ulm, Germany

**Keywords:** Chemokine, CXCL13, Neurosyphilis, Lyme neuroborreliosis, CSF, Multiple sclerosis

## Abstract

**Background:**

The chemokine CXCL13 has been discussed as a diagnostic parameter with high specificity for Lyme neuroborreliosis (LNB) and as a marker of disease activity. Neurosyphilis and LNB share similar characteristics. We investigated retrospectively CXCL13 levels in the cerebrospinal fluid (CSF) of patients with neurosyphilis at initial diagnosis and during treatment.

**Results:**

Five patients with neurosyphilis were identified retrospectively using an electronic database in a tertiary care hospital from 2005 to 2012. CXCL13 levels were measured using an ELISA. Five patients with definite LNB and 10 patients with multiple sclerosis (MS) served as controls. Median CXCL13 levels at baseline were 972 pg/mL for neurosyphilis patients, 8,000 pg/mL for LNB patients, and 7.8 pg/mL for MS patients. Patients with LNB and neurosyphilis showed significantly higher CXCL13 levels in their CSF compared to MS patients (p < 0.05, p < 0.001, respectively). CXCL13 levels in the CSF declined during treatment.

**Conclusion:**

CXCL13 levels in the CSF of patients with neurosyphilis can be as high as in patients with LNB, exceeding the proposed threshold of 250 pg/mL for the diagnosis of LNB. Patients with encephalitic/myelitic syndromes appear to have especially high levels of CXCL13. Clinicians should be aware that high levels of CXCL13 are not found exclusively in LNB but also in other infectious diseases of the CNS.

## Background

Syphilis is a sexually transmitted spirochetal disease caused by *Treponema pallidum* subspecies *pallidum*. The incidence of syphilis has been on the rise in western countries over recent years, and late manifestations of the disease occur with an incidence of approximately 7/100,000 inhabitants in the United States [1]. A more frequent occurrence of patients with neurosyphilis is to be expected in the coming years [2]. The chemokine CXCL13 is produced by antigen-presenting cells and is thought to play a role in attracting B cells and B-helper T cells into the cerebrospinal fluid (CSF) in neuroinfectious diseases [3]. CXCL13 is expressed at high levels in the CSF of patients with Lyme neuroborreliosis (LNB) [4–7]. The sensitivity and specificity of CXCL13 for the diagnosis of LNB are reported to be above 90% [4, 8]. Other non-infectious inflammatory diseases of the nervous system, like multiple sclerosis (MS), show only slight elevations of CXCL13 levels in the CSF [9]. In addition, CXCL13 levels in the CSF seem to be a surrogate marker for disease activity in LNB as CXCL13 levels correlate with pleocytosis and quickly fall after the initiation of antibiotic treatment [10, 11]. Despite these compelling findings, the specificity of CXCL13 for LNB is a matter of controversy, especially when other subacute diseases of the nervous system are considered. Elevated levels of CXCL13 were also reported for CNS lymphoma, HIV infection, cryptococcosis, and neurosyphilis [9, 12–14]. A cut-off at 250 pg/mL was thus proposed by van Burgel et al. [9] to distinguish between LNB and other diseases. However, there is no generally accepted cut-off for diagnosing LNB on the basis of CXCL13 levels in CSF. As neurosyphilis shares some similarities with LNB (e.g., spirochetal origin, subacute course, and similar CSF changes), we aimed to measure CXCL13 levels in the CSF of patients with clinically and serologically definite neurosyphilis before and during antibiotic treatment and compared the results with those of patients with definite LNB and MS.

## Methods

Using an electronic database search in a tertiary care university hospital (Albert Ludwigs University Freiburg), we retrospectively searched for patients with neurosyphilis from 2005 until 2012. Patients with definite neurosyphilis were diagnosed according to the criteria of the Centers of Disease Control and Prevention and the German Academy of Neurology (Deutsche Gesellschaft für Neurologie). Accordingly, they had to fulfill all of the following criteria: compatible neurological syndrome, CSF pleocytosis [white blood cell (WBC) count >5/µL], dysfunction of the blood–CSF barrier [elevated albumin CSF–serum quotient (Q_Alb_) and total CSF protein], positive Venereal Disease Research Laboratory (VDRL) test in CSF, positive *T. pallidum* particle agglutination assay (TPPA) in serum and CSF, and fluorescent *Treponemal* antibody-absorption (FTA-ABS) test in serum [15, 16]. Patients were excluded if they had relevant co-infections that could possibly interfere with the measurement of CXCL13 (e.g., HIV) or were treated with antibiotics before lumbar puncture. Patients with LNB had to fulfill the criteria proposed by Stanek et al. [17] and were matched with neurosyphilis patients for age and sex. MS patients were diagnosed according to the 2010 revised McDonald criteria [18]. They served as a “negative” control group and were not matched for age and sex. CSF samples were collected at the time of diagnosis and during the course of the disease as part of the initial diagnostic work-up via lumbar puncture. Superfluous CSF material was stored for further investigation at −80°C. CSF samples were stored, treated, and analyzed in the same manner for all included patients. CXCL13 levels were measured using an ELISA test system (Quantikine; R&D Systems, Minneapolis, MN, USA) according to the instructions supplied by the manufacturer. Details on the application of the assay for CSF have previously been published [4]. When follow-up CSF samples were available for the neurosyphilis patients, they were also used to measure CXCL13 in order to investigate the role of the chemokine in the course of the disease.

The study was approved by the Ethics Committee of the University Medical Center Freiburg, and written informed consent was obtained from all patients.

For comparisons between multiple groups, the Kruskal–Wallis test with Dunn’s post-test was used. For comparisons between two groups, the Mann–Whitney test was performed. Correlations were investigated with Spearman’s rank correlation coefficient. A *p* value of <0.05 was regarded as statistically significant. Statistics were performed with Prism 4.0b for Macintosh (GraphPad Software, San Diego, CA, USA).

## Results

Using our strict inclusion criteria, five consecutive patients with confirmed neurosyphilis were identified. The clinical, demographic, and CSF routine laboratory characteristics of these patients are shown in Table 1. All neurosyphilis patients had late-stage neurosyphilis. The clinical syndromes were heterogeneous. Subsequent to the first lumbar puncture, patients #1, #3, and #5 were treated with 30 Mio IU penicillin G IV for 14 days while patient #2 was treated with 24 Mio IU penicillin G IV for 14 days. Patient #4 was treated with 24 Mio IU penicillin G IV for 21 days and received additional treatment with corticosteroids to avoid occlusion of the cerebral vessels.

**Table 1:** Clinical and CSF parameters

Subject #	Age (years)	Sex	Clinical syndrome	CSF WBC count (/μL) (Ref.: <5)	Protein CSF (mg/dL) (Ref.: <450)	Q_Alb_ × 10 (Ref.: <8)	VDRL (CSF) (titer) (Ref.: <1)	CXCL13 in CSF (pg/mL) (Ref.: <10)		
								Baseline	1 month	3 months
1	79	M	Encephalomyelitis	18	878	9.3	2	292.6	66.8	17.7
2	55	F	Multiple cranial nerve palsies	25	933	12.5	8	134	24	7.8
3	56	M	Encephalomyelitis, cranial nerve palsies	42	648	6.3	32	6,600	183	n.a.
4	51	M	Cerebral vasculitis	192	1,160	12.4	32	972	184	n.a.
5	39	M	Meningoencephalitis	13	2,150	17.5	4	7,140	138	10

Clinical, demographic, and CSF routine laboratory characteristics at baseline and CXCL13 levels in CSF at baseline and during follow-up in neurosyphilis patients. Venereal disease research laboratory (VDRL) titers >1 are regarded as positive.

*WBC* white blood cell, *Q*_*Alb*_ albumin CSF–serum quotient, *n.a.* not available, *ref* reference values.

The mean WBC count in the CSF at the baseline level was 58/µL (SD 68, range 13–192) in patients with neurosyphilis, 209/µL (SD 138, range 84–427) in patients with LNB, and 5/µL (SD 4.5, range 1–14) in patients with MS. The mean total protein in the CSF at the baseline was 1,154 mg/L (SD 524, range 648–2,150) in patients with neurosyphilis, 1,445 mg/L (SD 711.7, range 977–2,650) in patients with LNB, and 491 mg/L (SD 139, range 345–743) in patients with MS.

The median baseline CXCL13 levels in the CSF samples were 972 pg/mL (IQR 213–6,870, range 134–7,140) for patients with neurosyphilis, 8,000 pg/mL (IQR 4,170–20,747; range 3,247–29,110) for patients with LNB, and 7.8 pg/mL (IQR 7.8–20.6; range 7.8–59.4) for patients with MS. The baseline levels of CXCL13 in patients with neurosyphilis showed a high variance, with the highest values observed in patient #5 with meningoencephalitis (7,140 pg/mL) and in patient #3 with encephalomyelitis (6,600 pg/mL).

**Figure 1:**
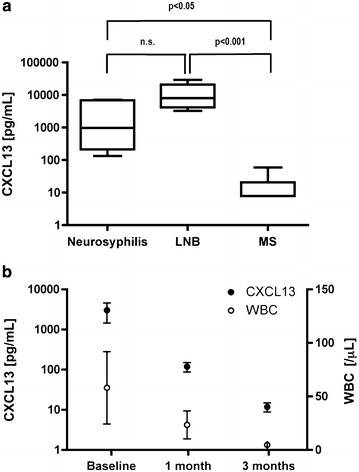
**a** CXCL13 levels in CSF at baseline in patients with neurosyphilis (n = 5), neuroborreliosis (n = 5, LNB), and multiple sclerosis (n = 10, MS). The *box* includes all values from the first (25%) to the third quartile (75%) while the *band inside the box* corresponds to the median. The ends of the *whiskers* represent the range. **b** CXCL13 levels (*left ordinate*) and WBC count (*right ordinate*) in the CSF of neurosyphilis patients during the course of disease. *Circles* represent the mean, and *whiskers* represent the standard errors. Correlation with Spearman‘s rank correlation: r = 1.00; *p* = 0.33.

There was a non-significant trend toward higher baseline values of CXCL13 in patients with LNB compared to neurosyphilis (Figure 1a). However, the difference in the mean baseline CXCL13 levels was statistically significant for both neurosyphilis and LNB compared to MS (Figure 1a, *p* < 0.05, *p* < 0.001, respectively). During the course of treatment, CXCL13 levels rapidly declined in neurosyphilis patients, paralleling the course of WBCs in the CSF (Figure 1b).

## Conclusions

This is the first controlled study reporting on CXCL13 levels in CSF from patients diagnosed with neurosyphilis but without confounding co-infections at the time of the first diagnosis and during the course of treatment. The presented results stem from a small population, therefore, the conclusions are rather limited. It may be noted that long periods of storage can lead to decreased CXCL13 levels. However, as some of the highest values of CXCL13 were estimated in the oldest samples, this issue seems unlikely. Schiffer et al. [19] report no discernible loss of CXCL13 after repeated cycles of freezing and thawing serum samples. Our main finding is that CXCL13 levels are substantially elevated in CSF from patients with untreated neurosyphilis and can surge as high as previously reported for patients with LNB [13]. Furthermore, the CXCL13 baseline values from 80% of our patients with neurosyphilis exceed the threshold of 250 pg/mL for the diagnosis of LNB proposed by van Burgel et al. [9]. However, there is no generally accepted cut-off for diagnosing LNB on the basis of CXCL13 levels in CSF as reported cut-offs range from <100 to more than 1,000 pg/ml [6, 7]. Whether a general cut-off for diagnosing LNB would be reasonable remains doubtful in the context of our finding that the CSF of patients with neurosyphilis can also show high levels of CXCL13. Other cytokines, such as neopterin, that are elevated in the CSF of LNB patients, but not in patients with other neuroinfectious conditions, are also elevated in the CSF of neurosyphilis patients [5]. Our results are in line with Marra et al. [20] who report similar CXCL13 levels in the CSF of 83 patients with HIV and symptomatic or asymptomatic neurosyphilis. However, HIV may also lead to elevated levels of CXCL13 in CSF, so the results of Marra et al. may not solely be due to neurosyphilis [9].

Levels of CXCL13 in the CSF of patients with neurosyphilis declined during antibiotic therapy, in parallel with the clinical improvement of symptoms and a decrease in the WBC count in the CSF, thus representing another marker of CNS inflammation. CXCL13 levels might be part of the inflammatory response to infection of the CNS with *T. pallidum* and may serve as a marker of disease activity in neurosyphilis. Marra et al. [20] report declining CXCL13 levels during treatment which is corroborated by our findings. Other reports have shown different CXCL13 levels in the CSF of patients with neurosyphilis, which were found to be remarkably lower than in CSF samples from patients with LNB [9, 14, 21]. This difference could be due to pre-analytical sampling and handling conditions. Different clinical manifestations of neurosyphilis may account for the diverging results. In our study, neurosyphilis patients with encephalitic and/or myelitic syndromes (e.g., spastic gait, seizures, or aphasia) showed the highest CXCL13 levels in their CSF. Both patients with isolated palsies of cranial nerves or cerebral luetic vasculitis showed substantially lower CXCL13 levels. Unfortunately, Rupprecht et al. and van Burgel et al. provide no information on the clinical syndromes. Marra et al. do not provide details on the clinical manifestations of neurosyphilis in their patient population but show that CXCL13 levels are higher in patients with HIV and neurosyphilis compared to patients with HIV and syphilis but without neurological symptoms. A possible association between different neurological manifestations and CXCL13 levels in CSF should be the subject of further prospective investigations in neurosyphilis.

In conclusion, as elevated CXCL13 levels are not pathognomonic for a single condition, interpretation of CSF analysis should only be performed by including measurements of antibodies against both *Borrelia burgdorferi* and *T. pallidum* in CSF. Clinicians should be aware that high levels of CXCL13 are not found exclusively in LNB but in various infectious diseases of the CNS.

## Abbreviations

CSF: cerebrospinal fluid
LNB: Lyme neuroborreliosis
MS: multiple sclerosis
Q_Alb_: albumin CSF–serum quotient
VDRL: venereal disease research laboratory
WBC: white blood cells
